# A randomized controlled trial of ultrasound-assisted technique versus conventional puncture method for saphenous venous cannulations in children with congenital heart disease

**DOI:** 10.1186/s12871-021-01349-y

**Published:** 2021-04-27

**Authors:** Yong Bian, Yanhui Huang, Jie Bai, Jijian Zheng, Yue Huang

**Affiliations:** 1 Department of Anesthesiology, Shanghai Children’s Medical Center Affiliated to School of Medicine, Shanghai Jiao Tong University, 1678 Dongfang Road, Pudong, Shanghai, 200127 China; 2 Department of Anesthesiology and Pediatric Clinical Pharmacology Laboratory, Shanghai Children’s Medical Center Affiliated to School of Medicine, Shanghai Jiao Tong University, Shanghai, China

**Keywords:** Congenital heart disease, Ultrasound-assisted technique, Conventional puncture method, The saphenous vein

## Abstract

**Background:**

The study investigated the success rate of the great saphenous venous catheter placement performed by ultrasound-assisted technique compared with the conventional puncture method in infants and toddlers with congenital heart disease and aimed to assess the efficiency and feasibility of this method within the context of pediatric peripheral venous access.

**Methods:**

We selected infants and toddlers who underwent congenital cardiac surgery in our medical center from June 1, 2020, to September 7, 2020, by convenience sampling. Children were stratified by the presence of the manifesting cardiac types (cyanotic or acyanotic heart disease). They were assigned to the conventional puncture method group or the ultrasound-assisted group through randomly blocked randomization. The primary outcome was the success rate of the first attempt. The second outcomes included the time to cannulation at the first attempt, the redirections of the first attempt, overall puncture time, and overall redirections of efforts. Besides, a binary logistic regression model was implemented to identify the possible variables related to the success rate of the first attempt.

**Results:**

A total of 144 children in our medical center were recruited in the study. The success rate of the first attempt in the ultrasound-assisted group was higher than that of the conventional puncture method group in the stratification of cyanotic children (66.7% vs. 33.3%, *P* = 0.035). Among children of acyanotic kind, the difference in the success rate of the first attempt between the two groups was not significant (57.6% vs. 42.4%, *P* = 0.194). Overall puncture time (45.5 s vs. 94 s, *P* = 0.00) and the time to cannulation at the first attempt (41.0 s vs. 60 s, P = 0.00) in the ultrasound-assisted group was less than the conventional puncture method group. The ultrasound-assisted group also required fewer redirections of the first attempt (three attempts vs. seven attempts, *P* = 0.002) and fewer total redirections of efforts (two attempts vs. three attempts, *P* = 0.027) than the conventional puncture method group. The result of binary Logistic regression showed that the success rate of the first attempt was related to age (OR:1.141; 95% CI = 1.010–1.290, *P* = 0.034), the redirections of the first attempt (OR:0.698; 95% CI = 0.528–0.923, *P* = 0.012) and the saphenous venous width (OR:1.181; 95% CI = 1.023–1.364, *P* = 0.023).

**Conclusions:**

The ultrasound-assisted technique improves the saphenous venous cannulation sufficiently in children with difficult peripheral veins. The younger age is associated with a higher likelihood of peripheral venous difficulty. The ultrasound-assisted methods can effectively screen peripheral veins, e.g., selecting thicker diameter peripheral veins, making puncture less uncomfortable, and improving success rates. This method can be used as one of the effective and practical ways of peripheral venipuncture in children, especially in difficult situations. It should be widely applied as one of the alternative ultrasound techniques in the operating room.

**Trial registration:**

ChiCTR.org.cn (ChiCTR-2,000,033,368). Prospectively registered May 29, 2020.

## Background

Peripheral intravenous cannulation in pediatric cardiac surgery is a standard invasive procedure performed for fluid-infusion or anesthetic care in the operating room (OR). For the potential instability of children with congenital heart disease (CHD), the practical and rapid establishment of peripheral venous lines in the OR is critical. Delayed venous access may lead to unexpected adverse events. The success rate for peripheral venipuncture had been reported with varying results in infants and toddlers using the conventional puncture method, with the overall success rate ranging from 30 to 64% [1–3]. Considering the characteristics of poor nutritional status, younger age, and lousy peripheral venous circulation, obtaining venous access can be challenging in children with cardiac anomalies, among which cyanotic children are, in particular, tough to deal with [4–6]. Also, several articles [7, 8] had reported an apparent high prevalence of Ehlers-Danlos syndrome, a severe heritable disorder of connective tissue in complex congenital heart defects, which may further deteriorate the peripheral venous conditions. The most commonly selected peripheral veins for infants in clinical maneuvers include dorsal hand veins, antecubital veins, and saphenous veins. Due to its superficial location and anatomical positioning, the saphenous vein has gradually become one of the preferred lines of cannulation for many anesthetists [9].

In recent years, ultrasound-guided venipuncture has attracted extensive attention attributed to its visualization and minor harm features. Its effectiveness in central venipuncture and arterial puncture had been demonstrated consistently in the literature [10, 11]. The significance of this technique in peripheral veins, especially in infants, continues to be controversial. Benkhadra M et al. [12] found that the use of ultrasound after anesthesia effectively reduced the number of punctures of peripheral veins and the time of successful cannulation in infants. However, Aaron E et al. [13] indicated that ultrasound-guided peripheral venipuncture did not reflect its advantages with limited ultrasound training. Besides, most previous studies about the sonographic application focused on the emergency department (ED) population. In contrast, less attention had been given to perioperative children, especially with congenital cardiac anomalies.

Therefore, in this study, we performed a randomized clinical trial to evaluate the success rate of the first attempt and the overall number of punctures for great saphenous vein cannulation with the use of the ultrasound-assisted technique compared with the conventional puncture method in infants and toddlers with congenital heart disease. We hypothesized that this technique would improve the efficacy of peripheral venous access in this population.

## Methods

### Study design and population

This study was a prospective stratified permuted block randomized controlled trial that compared the great saphenous vein’s puncture effect using the ultrasound-assisted technique with the conventional puncture method in the exclusive group of children with congenital heart defects. Eligible participants undergoing cardiac surgery in our hospital were recruited from June 2020 to September 2020. Ethics approval for the study was obtained by the Institutional Review Board of Shanghai Children’s Medical Center (approval number: SCMCIRB-K2020049–1). The whole trial protocol was performed in accordance with the Declaration of Helsinki. Also, the study protocol was registered at http://www.chictr.org.cn (number: ChiCTR2000033368; principal investigator: Yue Huang; date of registration: May 29, 2020). The ultrasound equipment used in the study was licensed for clinical application.

### Study protocol

We included participants from a convenience sample of all eligible children requiring congenital cardiac surgery. Infants and toddlers aged 0 ~ 3 years, with ASA scores of I-III and congenital heart disease, were all enrolled. Exclusion criteria included infection or hemangioma at or near the puncture site within one month previously, recent great saphenous venous puncture history, ASA score > III, or an emergency surgery needed. The medical history was collected, including age, sex, height, weight, previous history of difficult access, comorbidity, and types of cardiac anomalies. Informed consent was obtained from parents or guardians of all children after the anaesthesiologists in the study explained the study protocol. Children were assessed for inclusion and were randomly assigned to the routine conventional puncture method group or the ultrasound-assisted group at the level of types of a pre-existing cardiac anomaly, including cyanotic or acyanotic congenital heart disease. Cyanotic congenital heart disease was defined as the presence of SpO2 < 85% at rest or after crying without oxygen supplement and coexisting mixing intracardiac pathology (e.g., right-to-left shunts at the atrial or ventricular level). Acyanotic congenital heart disease was defined as intracardiac pathology (e.g., left-to-right shunt at the atrial and ventricular levels) with SpO_2_ ≥ 85% in both static and crying conditions. The random numbers were automatically generated through the Excel operating program. A balanced Stratified block randomization assignment was adopted to ensure the equal size of comparison groups throughout the study. We used fixed block sizes of four with an overall block of 18 and an allocation ratio of 1:1 separately. The corresponding random numbers were kept in sealed, opaque envelopes, which were employed in sequence according to the order of patient presentation. Children with cyanosis have the features of cardiovascular instability and poor peripheral circulation caused by long-term hypoxia. So this study was expected to find differences in the success rate of puncture between the subgroups. To reduce the influence of operators’ puncture technique on the outcome judgment, the procedures in this study were performed only by one anesthesiologist of our hospital with 25 years of experience in anesthesia. Before the study, The anesthesiologist underwent theoretical training of ultrasound-assisted technique in the details of ultrasonic machine operation and image analysis by ultrasound professionals. Apart from theoretical training, a hands-on simulation of artificial limbs’ real-time process was carried out to help the practitioner master this skill. Besides, clinical practice with the ultrasonic device was carried out for two weeks before the trial started.

**Fig. 1:**
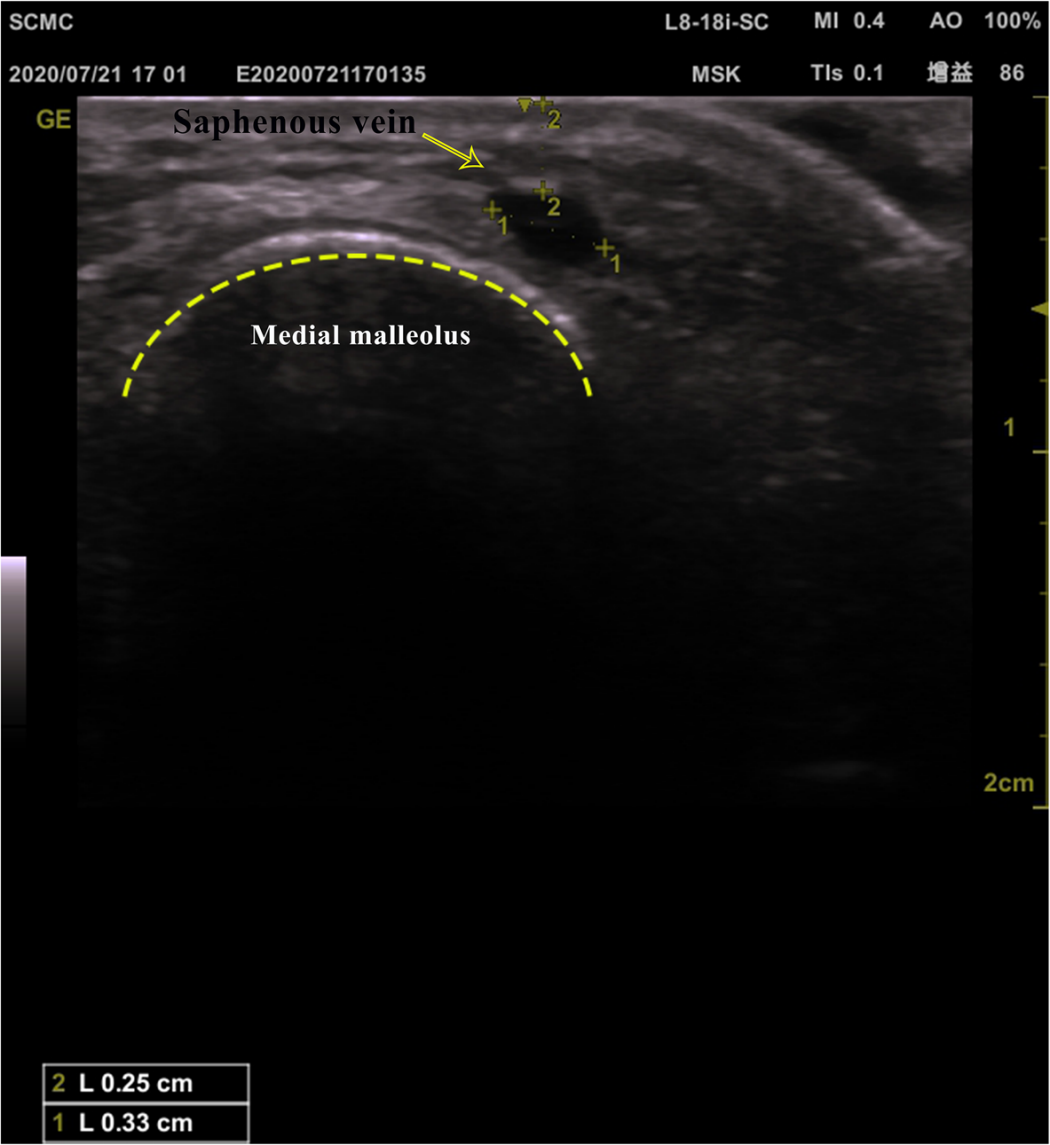
Legend of ultrasonic measurement of depth and width of a saphenous vein, symbol 1 is the width; symbol 2 is the depth

Before getting into the operating room, the child was given 0.5 mg/kg midazolam orally by the sedative nurse. If Ramsay score ≥ 4 were met, the child would be brought into the operating room, and random numbers were extracted from the envelope to determine the group. If the participant did not sleep during the first 30 min, 1 μg/kg of dexmedetomidine would be added to avoid separation anxiety for the child and family members. After entering the operating room, the child’s saphenous venous condition was graded using the venous grading criteria by the anesthesiologist conducting the venipuncture in this experiment. The grading criteria are shown in Table 1. The vein distance from the top of the vein to the skin (depth) and the maximum transverse vein diameter (width) of the bilateral saphenous vein at the level of medial malleolus were then measured by another anesthesiologist with extensive ultrasound experience using an 8–18 MHz Linear probe of a GE Healthcare ultrasound device (Venue 50, GE Healthcare) and the ultrasonic depth was standardized to 2 cm. The depths and widths of the vein in the same short-axis plane were measured three times to determine the final parameter by the average values of the measurements. The measurement legend of the great saphenous vein was shown in Fig. 1. All procedures were performed using water-soluble ultrasound transmission gel as a contact medium. The corresponding images were saved so that the operator could review the picture and the measurement results of the saphenous vein selected by the operator afterward.

**Table 1:** The grading criteria of saphenous vein punctures

I Visible bilateral saphenous veins, no difficulty in puncture	
II Only a saphenous vein visible, predictable difficult access	
III No visible or palpable bilateral saphenous veins

The ultrasound-assisted IV catheter placement was performed using a static ultrasonic technique. Briefly, the operator was permitted to apply a tourniquet to the proximal part of the ankle. For the ultrasound group, the operator performed ultrasonic equipment to scan the entire field of both saphenous veins in the short axis view to select the better one for the cannulation with the tourniquet applied. In two points with 1 cm distance to each other in the short-axis plane, the overlying skins were marked with a straight line using a black marker. Once the puncture site was identified and the vein marked, the field was prepared aseptically. Then the operator immediately used the skin marking as a landmark for subsequent IV access attempts. A cannula was chosen at the discretion of the study operator. Once a vein was selected, the operator was not permitted to change sides during the study. When a flash of blood was seen in the trocar, that is, the cannula penetrated the anterior wall of the vein, the needle angle was lowered, and afterward, the cannula was advanced into the vein over the needle. In contrast, for the conventional group, after the tourniquet was applied, the choice of the great saphenous vein was made by the experience of the operator, which included the visualization and the palpation of the great saphenous vein. After the puncture site was identified, regular disinfection was made. The operator performed the catheterization just anterior to the medial malleolus of the ankle. Subsequent operations were similar to the ultrasound group. If the first attempt failed, the choice of following puncture location was left at the discretion of the anesthetist. The overall puncture time and other parameters were recorded during the puncture process, and the specific definitions are described in the upcoming section. The overall puncture time and other parameters were recorded and calculated with a stopwatch by an anesthesiologist who was not involved in the anesthetic care of the child, and results were self-reported. The anesthesiologist was strictly trained about the procedure and time calculation to ensure the accuracy of the record. To avoid unnecessary body movement during puncture, inhaled sevoflurane (5%) in oxygen at 5 L/min was delivered to the child via a face mask. If the minimum alveolar concentration (MAC) reached 0.8, the puncture was performed. Standard monitoring was applied throughout the period, including blood pressure, heart rate, and pulse oxygen saturation. At the end of the surgery, the complications of the puncture site were recorded, such as skin swelling and whiteness, venous extravasation, hematoma. Because of the physical nature of the interventions, it was not possible to conceal the group allocation from the research assistants or the trial operator. Still, the random numbers were maintained separately by a nurse who was not involved in the study and data processing and was not revealed to the research investigators and statisticians until data entry and analyses were completed.

### Outcome measurements and definitions

We recorded the age, sex, height, body weight, Previous history of difficult access, and any comorbidities that might render IV access difficult, including the chromosomal abnormality, severe intellectual disability, and the types of the cardiac anomaly. BMI was measured by height and weight (BMI = weight/(height) ^2^).

The primary outcome was the success rate of the first attempt in either group. The successful venous cannulation was defined as catheter placement with reflux of blood into the catheter and subsequent ability to infuse 5 mL of standard saline flush without local infiltration. If the insertion of the first attempt was not successful, the procedure was considered as a failure of the first attempt. Unsuccessful venous cannulation after three attempts was described as a failure of overall punctures. The secondary outcomes included the overall numbers of attempts, the overall puncture time, overall numbers of needle redirections, the time to cannulation at the first attempt, numbers of needle redirections at the first attempt. One attempt was described as a puncture in which the needle enters the skin directly for intravenous catheterization, and the endpoint of an attempt was the withdrawal of the needle out of the skin. The time of one puncture was identified as the time starting from tourniquet placement to the end of confirmation of the flush of normal saline. The overall puncture time was calculated by accumulating the time of each puncture. A redirection was defined as the partial withdrawal of the catheter with subsequent advancement to change the direction of catheter placement. The numbers of needle redirections at the first attempt were counted. The overall numbers of needle redirections were calculated by accumulating total needle redirections of attempts. If the needle was redirected more than six times, no further adjustment would be made to avoid harm to children.

### Statistical analysis and sample size calculation

All analyses in this study were performed on an intention to treat basis. Statistical package for social sciences (SPSS) version 24 for windows (SPSS Inc., Chicago, IL, USA) was used in the analysis of the data obtained. The normality of continuous data was tested by the Shapiro-Wilks method. Means ± standard deviations were computed for the continuous and normally distributed data. A two-sided independent sample t-test was used for intergroup comparison. Skewed distribution data were presented as medians with ranges. The Mann-Whitney tests were used for Skewed distribution data and ordered categorical variables. Proportions and associated 95% confidence intervals (CIs) were computed for categorical variables. Pearson chi-squared test or Fish’s exact test was used for unordered categorical variables, and relative risks and differences in proportions (with 95% confidence intervals (CIs) were computed between groups to compare the difference. When taking into account the confounding factor (the types of cardiac anomaly), the Cochran-Mantel-Haenszel method was used for stratified data. A *P*-value of less than 0.05 was considered statistically significant. A multivariate logistic regression model was performed to test if the success rates at the first attempt were affected by possible confounders such as types of the cardiac anomaly, the puncture methods selected, age, sex, BMI, previous history of difficult access, comorbidities, saphenous veins grading, numbers of needle redirections at the first attempt, the diameter and depth of the saphenous veins. All variables above were analyzed firstly by univariate logistic regression. If P was less than 0.2, the variable was included in the multivariate logistic regression model for correlation analysis. Associations between two continuous covariates were assessed by the Pearson correlation coefficient. If correlation coefficients were more than 0.8, the more statistically significant variable was selected in the multivariate logistic regression model to avoid multicollinearity among the potential covariates. Finally, Receiver operating characteristic (ROC) curve and Hosmer-Lemeshow test were used to assess the discrimination and calibration of the logistic model.

The sample size required was calculated on the basis of the success rate of the first attempt. The previous study reported first-attempt success rates of about 51% with the conventional method for saphenous venous cannulation [14]. We assumed that a 30% increase in the success rate of the first attempt using the ultrasound technique should be significantly different compared with the conventional approach. This assumption required 60 participants in each group to detect a difference in the success rate of the first attempt between groups with a power of 80% and a significance level of 0.05 using a two-sided Chi-square test. Taking into account the balance of the random block and the 10% dropouts of the samples, the final sample size was raised to 72 cases in each group. This sample size also allowed us to effectively find the effects of stratified factors (types of cardiac anomaly).

## Results

**Fig. 2:**
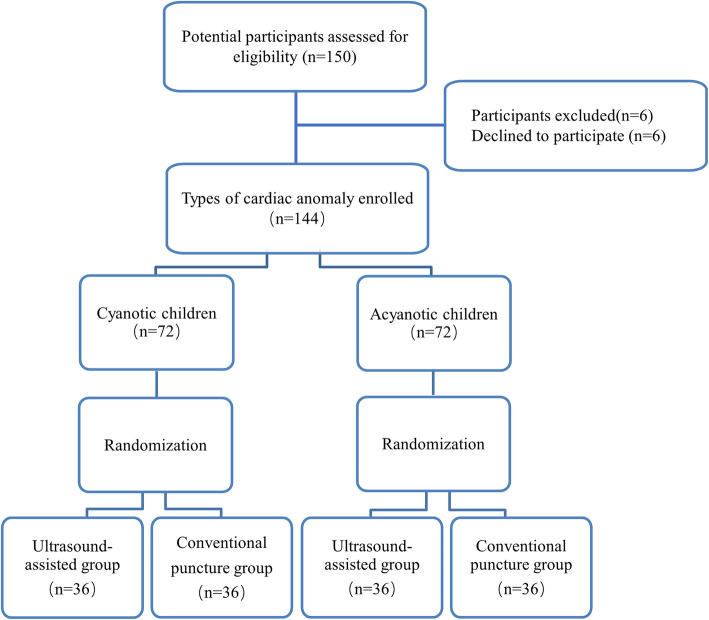
A flowchart of participants’ selection and allocation to study arms, no patients were dropped out of the study

From June 1, 2020, to September 7, 2020, a total of 150 children with congenital heart disease were approached for enrollment, with six excluded due to their parents’ refusal to participate. The remaining participants were enrolled, with 72 cyanotic children and 72 acyanotic children. A randomization scheme of the assigned arms of the trial is shown in Fig. 2. No case was dropped out during the study. Specific types of congenital heart disease were listed in Table 2. All data of enrolled children could be used for later statistical analysis. The demographic and baseline characteristics of the two groups were similar across the study arms (Table 3).

**Table 2:** Cardiac anomaly of patients enrolled

Cardiac anatomy	Count (n)
Cyanotic heart disease	
Tetralogy of Fallot	59
Pulmonary stenosis	12
Pulmonary atresia	1
Total	72
Acyanotic heart disease	
Ventricular septal defect (VSD)	34
Atrial septal defect (ASD)	7
Patent ductus arteriosus (PDA)	1
Mixed type (including VSD、ASD or PDA)	30
Total	72

**Table 3:** Baseline characteristics of study participants assigned to the ultrasound-assisted or the conventional puncture group to peripheral intravenous catheterization

	ultrasound-assisted group	. conventional puncture group	P-value
Age (month)	7(5–9)	7(4–9)	0.445
Sex (male/female)	48/24	43/29	0.449
BMI (kg/cm^2^)	16.31 + 1.94	16..56 + 2.24	0.186
Previous history of difficult access, %(Y/N)	15.3%(11/61)	16.7%(12/60)	0.803
Comorbidity, %(Y/N)	13.9%(10/62)	5.6%(4/68)	0.114
Venous grading(I/II/III)	5/13/54	4/11/57	0.509

*BMI* body mass index

The primary outcome result The success rate of the first attempt between the ultrasound-assisted group and the conventional puncture method group differed significantly (*P* = 0.017). Compared with the conventional group, the difference in the proportion of the success rate of the first attempt for the ultrasound group was 24.5%(95% CI: 8.6–40.4%). The Breslow-Day test showed significant evidence for heterogeneity of odds ratios (*P* = 0.042) on the level of types of a cardiac anomaly. After adjusting simultaneously for types of cardiac anomaly, the success rate of the first attempt was still associated with the choice of the puncture method using the Cochran-Mantel-Haenszel test (X^2^ = 4.841, *P* = 0.028). But when stratified by the types of a cardiac anomaly, among children with cyanosis, the success rate of the first attempt was much higher in the ultrasound group (66.7%) than in the conventional group (33.3%), and the difference was significant (*P* = 0.035). The difference in the proportion was 33.3%(95% CI: 11.6–55.1%). However, among children with no cyanosis, the difference in the success rate of the first attempt was not significant between the study arms (57.6% vs. 42.4%, *P* = 0.194). The difference in the proportion was 16.7%(95% CI: − 6.1-39.4%) (Table 4).

**Table 4:** Success rate of the first attempt in the ultrasound-assisted group versus conventional puncture group

	ultrasound-assisted group	Conventional puncture group	P-value
Total(n)	72	72	
Success on first attempt (n)	45	27	0.017
%(95% CI)	61.4(50.7, 73.3)	38.6(26.3, 48.7)	
Rate difference, %(95% CI)	24.5(8.6, 40.4)	–	
Relative risk, (95% CI)	1.547(1.072, 2.235)	0.640(0.436, 0.939)	
Cyanosis(n)	36	36	
Success on first attempt (n)	24	12	0.035
%(95% CI)	66.7(51.3, 82.1)	33.3(17.9, 48.7)	
Rate difference, %(95% CI)	33.3(11.6, 55.1)	–	
Relative risk, (95% CI)	1.714(1.042, 2.820)	0.545(0.293, 1.017)	
Acyanosis(n)	36	36	
Success on first attempt (n)	21	15	0.194
%(95% CI)	57.6(42.2, 74.4)	42.4(25.6, 57.8)	
Rate difference, %(95% CI)	16.7(−6.1, 39.4)	–	
Relative risk, (95% CI)	1.413(0.823, 2.428)	0.716(0.432, 1.187)	

*CI* confidence interval

The secondary outcome results The overall success rate was 90.3% in the ultrasound group compared with 77.8% in the conventional group(*P* = 0.08; difference 12.5%(0.7 to 24.3%)). The Breslow-Day test showed no significant evidence for heterogeneity of odds ratios (*P* = 0.103) on the level of types of a cardiac anomaly. The overall puncture time was 45.5 s (36 s to 96.25 s) in ultrasound group compared with 94.0 s (56 s to 171 s) in conventional group(*P* = 0.00); The time to cannulation at the first attempt was 41.0 s (35 s to 53.75 s) in ultrasound group compared with 60 s (45 s to 83 s) in conventional group(P = 0.00). Significant differences of measures above suggested that the overall puncture time and the time to cannulation at the first attempt in the ultrasound group were much shorter. Besides, there were significantly fewer overall numbers of attempts (*P* = 0.002), overall numbers of needle redirections(*P* = 0.001), and needle redirections at the first attempt(*P* = 0.027) in the ultrasound group compared with the conventional group. The width and depth of the saphenous veins measured between groups were not significantly different at (1.485 + 0.318) mm vs. (1.543 + 0.396) mm(*P* = 0.375, 3.622 + 1.408) mm vs. (3.605 + 1.421) mm (*P* = 0.949), respectively.

The complication was rare. In the ultrasound group, two children had venous extravasation, and one child had local pale skin after surgery, while venous extravasation also occurred in two children in the conventional group. Patient complication rates did not significantly differ between groups(*P* = 0.999). The cannula was removed in all cases with complications, and no further severe adverse event was observed. The results of the secondary outcome are summarized in Table 5.

**Table 5:** The secondary outcomes in the ultrasound-assisted group versus conventional puncture group

	ultrasound-assisted group	conventional puncture group	P-value
The overall proportion of success rate(n), %	65(90.3%)	56(77.8%)	0.080
Rate difference, %(95% CI)	12.5(0.7, 24.3)	–	
The overall time to cannulation(s)	45.5(36, 96.25)	94.0(56, 171)	0.00
The time to cannulation at the first attempt(s)	41.0(35, 53.75)	60(45, 83)	0.00
Overall puncture numbers	1(1, 2)	2(1, 3)	0.002
Overall numbers of needle redirections	3(1, 7)	7(3, 13)	0.001
Needle redirections at the first attempt	2(1, 5)	3(2, 6)	0.027
The width of saphenous veins (mm)	1.485 + 0.318	1.543 + 0.396	0.375
The depth of saphenous veins (mm)	3.622 + 1.408	3.605 + 1.421	0.949
Complication (n), %	3(4.27%)	2(2.78%)	0.999

**Fig. 3:**
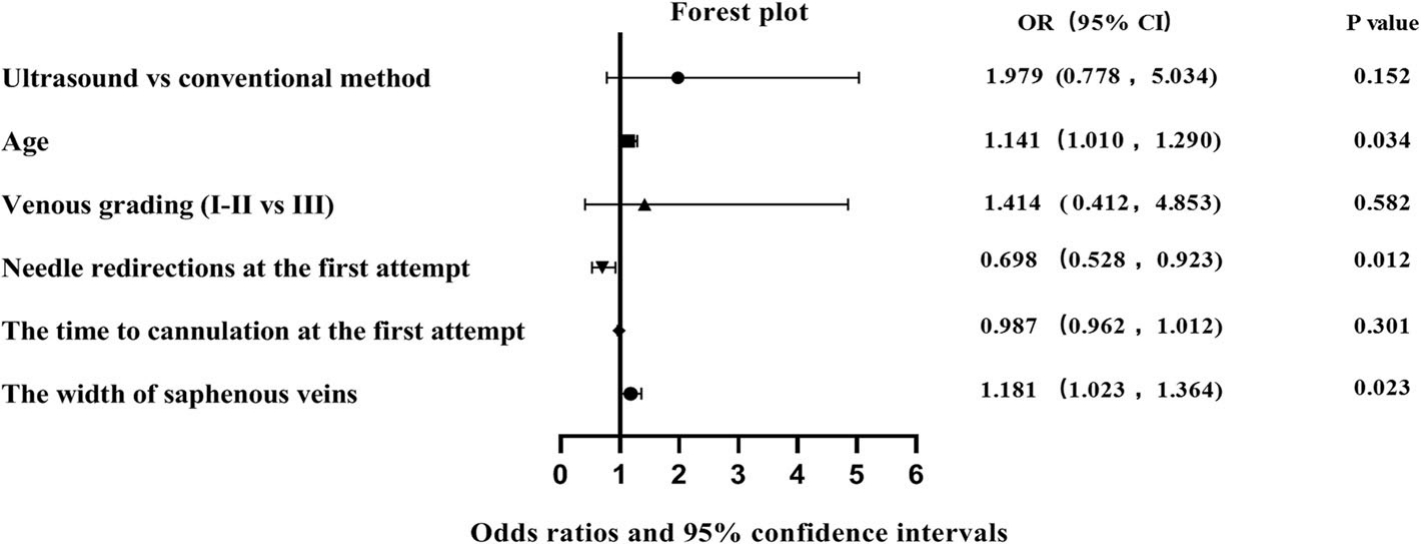
Forest plot of multivariate logistic regression analysis demonstrating independent factors associated with the success rate of the first attempts. Hosmer and Lemeshow *P* = 0.682

The potentially associated factors were screened by univariate logistic regression analysis to test their impact on the success rate of the first attempt. Due to the relatively small numbers of patients with grade I of venous conditions, grade I and II children were combined to decrease the possibility of inaccurate logistic modeling results. Six variables were selected to enter into the final multivariate logistic regression model, including the puncture method(*P* = 0.019), age(*P* = 0.002), venous grading(*P* = 0.003), needle redirections at the first attempt(*P* = 0.000), the cannulation time of the first attempt(*P* = 0.001) and saphenous venous width(P = 0.003). Enter method was used to deal with variables in the multivariate logistic regression model, and the model revealed that the age (OR:1.141; 95% CI = 1.010–1.290, *P* = 0.034), needle redirections at the first attempt (OR:0.698; 95% CI = 0.528–0.923, *P* = 0.012) and saphenous venous width (OR:1.181; 95% CI = 1.023–1.364, *P* = 0.023) were associated with the success rate of the first attempt. The results of binary logistic regression are shown in Table 6. The data used in the multivariate model are also visualized as a Forest plot (Fig. 3).

**Table 6:** Results of the multivariate logistic regression model to examine factors associated with the success rate of the first attempt (*N* = 144)

Variables	β	OR(95% CI)	P-value
Puncture method			0.152
Ultrasound-assisted	0.683	1.979 (0.778, 5.034)	
Conventional puncture	Reference	Reference	
Age	0.132	1.141(1.010, 1.290)	0.034
Venous grading			0.582
I-II	0.346	1.414 (0.412, 4.853)	
III	Reference	Reference	
Needle redirections at the first attempt	−0.359	0.698 (0.528, 0.923)	0.012
The time to cannulation at the first attempt	−0.013	0.987 (0.962, 1.012)	0.301
The width of the saphenous veins	0.167	1.181 (1.023, 1.364)	0.023
Constant	−1.913	0.148	0.130

**Fig. 4:**
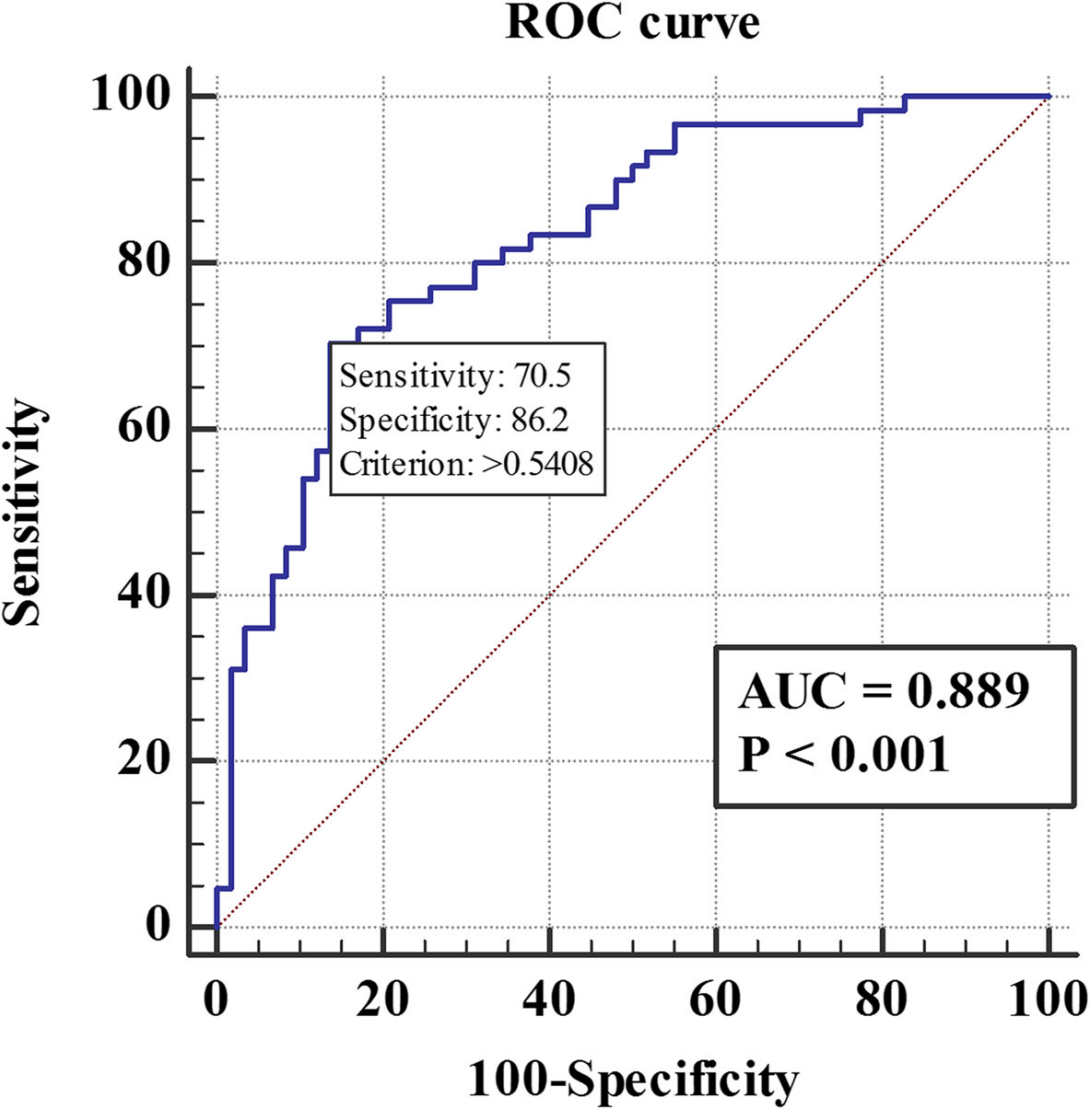
ROC curve for the multivariate logistic regression model. AUC:The area under the curve; AUC = 0.889, *P* < 0.001, The sensitivity and specificity are 70.5 and 86.2%, respectively

The area under the curve (AUC) for the ROC of the final model was 0.889(*P* < 0.01) (Fig. 4). Hosmer-Lemeshow goodness of fit test suggested the model fitted adequately (χ^2^ = 5.685, *P* = 0.682). Both of them showed good discrimination and calibration of the model.

## Discussion

Peripheral venous catheterization is one of the prime and critical steps of anesthesia in the operating room. Owing to the unique properties of this age group, such as thicker subcutaneous tissue, incompatibility to operation, and poor peripheral blood circulation after long-term fasting [15], infants and toddlers may present greater challenges with IV access. This could be especially tricky in younger children or those with complex illnesses such as congenital heart disease. The choice of suitable veins and puncture methods is the critical point to facilitate peripheral IV placement. In addition to its shallow position, the saphenous vein may offer other features such as the greater vessel diameter and relatively fixed location, which would make it a desirable option for IV placement for infants and toddlers [14]. Riera et al. [9] recommended saphenous veins as a superior first choice for IV cannulation in younger children. However, there could be no denying that even in the hands of a most experienced anesthesiologist, the failure of peripheral venous puncture remains high with the conventional approach. So improving the success rate of puncture of peripheral veins through the current accessible ultrasound-assisted technology is not only crucial for the improvement of the efficacy of anesthesia but also helpful for the reduction of costs and children’s discomfort related to multiple punctures. This may be especially pronounced in those being critically ill.

The present study demonstrates that the ultrasound-assisted technique is superior to the conventional approach for successful first-attempt saphenous vein cannulation in children with cyanotic congenital heart disease, whereas this effect did not occur in children with non-cyanotic congenital heart disease. Multiple factors like longtime chronic consumption, hemodynamic disability, and accompanying comorbidities entail potential difficulties in performing IV cannulation in children with cyanotic congenital heart disease. These may likely explain the difference. So we postulate that the precise localization of ultrasound allows the anesthesiologist to more accurately determine the position of a vein, thereby increasing the success rate. In contrast, in most situations, children with non-cyanotic congenital heart disease have good exposure of peripheral veins, which prevents the ultrasound method from showing its advantage of visualization compared with the conventional way. The success rate of the first attempt was used as one of the crucial metrics by many studies to evaluate the effectiveness of peripheral venipuncture [3, 16]. But no consistent conclusions have been drawn about the influence of the use of ultrasound on the success rate at the first attempt of peripheral venous access. In contrast to our results, Bair et al. [13] found that the use of static ultrasound-assisted technique could not increase the success rate of the first puncture of peripheral veins in young children. The main possible discrepancy may include 1. Participants enrolled in that study were derived from a pediatric ER setting, the circumstance of which may induce anxiety and fear of the children, and excessive body movement caused by these negative emotions could further make the venipuncture difficult; 2. Different types of patients and diseases of that study made it difficult to control the confounding factors completely; 3. The relatively small sample size (*n* = 44) increased the margin of error and compromised the conclusions drawn from the work. However, our research was a more extensive trial with participants in the operating room setting, and children were anesthetized without body movement. Also, only children with congenital heart disease were concluded. The above reasons may explain the difference between the two studies.

One study by Otani et al. [17] demonstrated US-guided IV placement using a real-time method led to a significantly lower IV success rate than the conventional technique in children with difficult IV access in the pediatric ED. Operators’ unfamiliarity with ultrasound technology and the demographic difference between groups may explain the inferior result. Nevertheless, a recent trial by Vinograde et al. [18] showed ultrasonographically guided intravenous line placement in children with predicted difficult intravenous access improved first-attempt success when conducted by a team of trained providers, which is comparable with our results. Another study by Rose et al. [19] also proposed more practice to improve the success rate of a difficult peripheral vein emphasizing the importance of experience. Moreover, the operator in this study had more than 20 years of experience in ‘blind’ peripheral venipuncture, whereas the ultrasound technique had only two weeks of hands-on experience before it was actually practiced. However, the success rate of the first attempt in children with cyanotic congenital heart disease using the ultrasound method was still higher than that using the conventional approach. So the fact of the high success rate of difficult access in the ultrasound group is vital from the point of view of the usefulness of ultrasound in peripheral venous invasive procedures. Based on the above results and analysis, the ultrasound-assisted technique outperforms the conventional approach on the saphenous vein cannulation for children with difficult peripheral veins (e.g., complex heart disease). In contrast, the effect of static ultrasound techniques in the general population of children may still require more investigation to determine between operators with different levels of experience.

Additionally, our results, in line with the previous studies [20], found that the overall puncture time and the first attempt time of the ultrasound-assisted group were shorter than that of the conventional approach group, and less numbers of redirection of the first attempt were needed in the ultrasound-assisted group. Considering the potential toxicity of general anesthesia in small children, all of these reductions are probably advantageous. The complications of peripheral vein puncture were generally rare, with only 5 cases in the study, three (4.27%) in the ultrasound group, and two (2.78%) in the landmark group. No statistical difference occurred between the complication rates according to the method used for the peripheral IV procedure. The complication rates are comparable to the previously reported result [21]. Both of the technique is relatively safe.

Inevitably, as the ultrasound-assisted technique is lack of real-time ultrasound guidance for the needle, the accuracy of this method is somewhat affected by the non-real-time availability of operations and may not be as high as that of the real-time ultrasound guidance technique. Many kinds of works [22, 23] had reported the effectiveness of a real-time ultrasound-guided method for peripheral venipuncture in children, while the efficacy of static ultrasound technique was less addressed. Munsey et al. [24] also analyzed in detail the advantages of dynamic ultrasound over static ultrasound in peripheral venipuncture. However, compared with the real-time ultrasound-guided venipuncture technique, the static ultrasound localization technique has its unique advantages of easy operation, a short learning curve period, and the absence of compression of the vein by the probe during puncture. Peripheral vein cannulation can be the most challenging in children, especially in small infants with poorly visible or palpable peripheral veins. Consequently, although recent guidelines by the European Society of Anaesthesiology [25] suggest the global use of ultrasound to assist all steps of the cannulation for central venous line placement in children, e.g., for internal jugular veins or femorally inserted central lines, they don’t recommend the routine use of ultrasound guidance for peripheral vein cannulation in pediatric patients. Simultaneously, the guidelines also addressed that the use of ultrasound may be of some benefit by experienced operators. Taking into account the relatively easy-to-operate and easier-to-grasp nature of the ultrasound-assisted technique, it also has particular application value in clinical practice, especially for ultrasound beginners. And the properties of this technique are even more crucial for anesthesiologists in developing countries. With insufficient ultrasound equipment, not every anesthetist has the opportunity to operate ultrasound equipment independently for long periods of time, which will definitely affect their proficiency in complex ultrasound techniques, such as the real-time ultrasound guidance technique. Sometimes with low-resolution ultrasound equipment, the operating of a more complex real-time ultrasound technique with two or more people coordinated could be embarrassing, especially if the baby is unable to cooperate. That was also the principal reason for us to choose this comparatively more straightforward technique in the study. Anyway, it was found from our research that in children with more difficult saphenous vein puncture, the ultrasound-assisted technique could effectively improve the success rate of the first puncture, but the difference was not found in less difficult cases like acyanotic children. So we hypothesize that with more training and practice, the use of a real-time ultrasound technique may further improve the success rate of the first puncture, even in children with no difficult peripheral veins. Thereby more investigations would be needed to prove the accuracy of both techniques on the basis of competence of skills. This aspect is one of our next-step research directions.

Multivariable logistic regression identified that the age, the width of saphenous veins was positively associated with the success rate of the first attempt, while the venous grading was negatively associated with the success rate of the first attempt. Schnadower et al. [26] and Hanada et al. [14] found that the diameter of the veins affected the success rate of the first puncture. Also, Carr et al. [27] found that age was one of the factors affecting the success rate of the first puncture through a multivariate regression model. All these studies are similar to our findings. The results of the model may provide some reference value for clinicians to access a difficult peripheral venipuncture in infants.

Our study has several limitations. First, the work is a single-center study with only one operator involved, which may limit the generalizability of the findings. A study by Anderson AP et al. [28] demonstrated that it took nine attempts after training for learners to achieve a 70% probability of success for ultrasound-guided peripheral intravenous catheter placement in pediatric patients. As for the larger samples in our research, the operator had more numbers of attempts to reach proficiency in the ultrasound technique and thus should be representative of a given population. But, puncture experience should have an impact on the success rate. It had been documented that using ultrasound-guided vascular equipment, experienced physicians had significantly higher success rates of peripheral venous punctures than inexperienced doctors [29]. Therefore, to further clarify the clinical significance of this technique, factors such as multiple study centers, larger samples, and physicians with different peripheral puncture experience should be added to prove its effectiveness and extensive nature. In addition, convenience sampling was used to recruit the participants in this study, rather than recruiting children’s samples consecutively. Although the sample size in this study was sufficient, there was still the possibility of sample selection bias. Lastly, although the static ultrasound technique has some inherent advantages, as we analyzed in the article, the real-time ultrasound technique may be more useful in cases where the operators are more experienced and skilled in the use of ultrasound techniques. However, the technique still has its clinical value, such as in cases of low resolution ultrasound equipments and less skilled ultrasound personnels.

## Conclusions

In our study, compared with the conventional puncture method, the ultrasound-assisted technique has the advantages of the higher success rate of the first attempt, shorter first puncture time, and fewer redirections for peripheral venous access. It should be effectively used as one of the reliable choices for peripheral venipuncture of children with difficult access, such as children with complex congenital heart disease. The younger age is associated with a higher likelihood of peripheral venous difficulty. The use of ultrasound technique can also screen suitable peripheral veins (e.g., choice of a larger diameter peripheral vein) to make localization more accurate with minor invasion and thereby increase the total success rate of peripheral venipuncture. Ultrasound technique for difficult peripheral venipuncture should be widely promoted in the operating room.

## Data Availability

The datasets generated during and analyzed during the current study are not publicly available due to the fact that some of the data in this study relate to the privacy of the children but are available from the corresponding author on reasonable request.
